# AntTrust: An Ant-Inspired Trust Management System for Peer-to-Peer Networks

**DOI:** 10.3390/s22020533

**Published:** 2022-01-11

**Authors:** Nehal Al-Otaiby, Afnan Alhindi, Heba Kurdi

**Affiliations:** 1 Department of Computer Science, College of Computer and Information Sciences, King Saud University, Riyadh 11451, Saudi Arabia; nfalotaiby@iau.edu.sa (N.A.-O.); aalhindi@ksu.edu.sa (A.A.); 2 Mechanical Engineering Department, Massachusetts Institute of Technology (MIT), Cambridge, MA 02139, USA

**Keywords:** peer-to-peer networks, trust management, wireless sensor networks, privacy

## Abstract

In P2P networks, self-organizing anonymous peers share different resources without a central entity controlling their interactions. Peers can join and leave the network at any time, which opens the door to malicious attacks that can damage the network. Therefore, trust management systems that can ensure trustworthy interactions between peers are gaining prominence. This paper proposes AntTrust, a trust management system inspired by the ant colony. Unlike other ant-inspired algorithms, which usually adopt a problem-independent approach, AntTrust follows a problem-dependent (problem-specific) heuristic to find a trustworthy peer in a reasonable time. It locates a trustworthy file provider based on four consecutive trust factors: current trust, recommendation, feedback, and collective trust. Three rival trust management paradigms, namely, EigenTrust, Trust Network Analysis with Subjective Logic (TNA-SL), and Trust Ant Colony System (TACS), were tested to benchmark the performance of AntTrust. The experimental results demonstrate that AntTrust is capable of providing a higher and more stable success rate at a low running time regardless of the percentage of malicious peers in the network.

## 1. Introduction

Computer networks, especially newly emerging forms such as wireless sensor networks (WSNs) and Internet of Things (IoT), are susceptible to failure [1,2,3]. Network failures can be attributed to a range of issues, of which security breaches are among the most common and dangerous. This is because, in these networks, a central component (e.g., gateway/router) is usually essential to the connection to the cloud or to the nearest network, which makes them more vulnerable, as a compromised central component can result in cascading failures. Therefore, many new application domains adopt the peer-to-peer (P2P) structure [4] or device-to-device cooperative (D2D) scheme [5], in which network nodes interact directly with each other, eliminating the need for the central component.

A P2P network is an open and dynamic distributed system, where nodes can directly communicate with each other without the need for a centralized server. In these networks, services are provided by peers; however, peers are characterized by their anonymity and freedom, so trust is essential to establishing communication among them [6]. Therefore, it is important to build a trust management system to encourage resource sharing among peers in such networks [7].

Many emerging studies have focused on trust management systems in P2P networks [8,9,10,11,12]. On the other hand, only a few studies have considered bioinspired approaches [13], although they might be of great benefit in such a context. Furthermore, to the best of our knowledge, all of the proposed bioinspired trust management algorithms are based on metaheuristics, also known as problem-independent heuristics, such as ant colony optimization (ACO) and artificial bee colony (ABC), rather than problem-specific heuristics. A problem-specific heuristic is a specifically tailored algorithm for a certain problem. Problem-specific heuristics are preferable to metaheuristics because they generate a “good” and inexpensive solution in terms of time and computing resources, unlike metaheuristics. A metaheuristic generates an optimal or near-optimal solution by extensively searching the solution space and iteratively generating candidate solutions before selecting the best among them, which is computationally expensive and time-consuming [14,15].

This paper proposes a novel trust management system, AntTrust, based on an ant-inspired problem-dependent heuristic. The proposed system helps to locate a trustworthy provider by mimicking the behavior of real ants in an ant colony. A new formula is proposed for the trust calculation between any two peers in the network based on four trust factors (trust value, recommendation, feedback received from other peers, and trust values collected from all friends in the community).

AntTrust was evaluated in a simulated P2P network model considering two types of attack strategies: malicious peers working alone, which are known as naïve malicious attacks, or malicious peers working in groups, which are known as collective malicious attacks. In our system, good peers are peers who always strive to provide valid files, correct feedback, and honest recommendations, while malicious peer models provide invalid files, incorrect recommendations, and dishonest feedback. AntTrust was benchmarked against three well-established trust management systems, namely, the TACS Trust Model for P2P Networks [8], EigenTrust [9], and the Trust Network Algorithm using Subjective Logic (TNA-SL) [16]. The results show the superiority of AntTrust, as it significantly increased the success rate of good peers at a remarkably low running time when compared to the other algorithms.

In summary, the main contributions of this paper are as follows:

- A novel trust management system, AntTrust, is proposed using a problem-specific heuristic to increase the success rate of good peers and reduce execution time.
- A reward formula is suggested to recompense peers that provide valid contents and honest recommendations.
- A new formula is introduced for the trust calculation between any two peers in the network based on four parameters: local trust value, recommendation, feedback, and friends’ trust values.
- A well-controlled evaluation framework is presented for evaluating the proposed approach.
- Validation of the proposed system is carried out against well-established trust management paradigms.

The paper is organized as follows: Section 2 reviews the main trust and reputation models related to this paper. Section 3 introduces the AntTrust algorithm, while Section 4 explains the evaluation methodology. The experimental results are presented and discussed in Section 5. Lastly, Section 6 summarizes the conclusions and suggests possible future directions of this research.

## 2. Related Work

Trust and reputation models can be classified into traditional systems and bioinspired systems. Traditional systems constitute any trust management system with a basis that does not stem from a biological system, e.g., EigenTrust [9] and TNA-SL [16]. In EigenTrust, each peer in the network is assigned a global trust value that can be calculated using the eigenvector. EigenTrust depends heavily on pre-trusted peers in trust calculations, making them the focal point of failures if overloaded or misled by malicious peers. To overcome this problem, HonestPeer [17] was introduced, where honest peers shoulder the load with pre-trusted peers. PeerTrust [11] considers several factors in trust calculations, such as feedback about a specific peer by other peers in the community, the number of transactions, the credibility of the feedback provider and transaction, and community context factors, weighted by the level of trust in the feedback provider. FuzzyTrust [18] is another way to handle trust. FuzzyTrust uses fuzzy inference to calculate local trust values and then aggregates these local values to produce a global reputation. PowerTrust [19] is a reputation system that utilizes a trust overlay network (TON). The system uses a regular random walk for the initial reputation value and a lookahead random walk for updating the reputation values from the power nodes. In [16], the Trust Network Analysis with Subjective Logic (TNA-SL) was proposed as a method based on subjective logic to evaluate the trust between peers in a P2P network. Trust is represented by the opinion of a peer about another peer. The opinion is a relationship between two peers and consists of four components: belief, disbelief, uncertainty, and base rate. TCR [20] classifies nodes with similar research topics into local subnetworks. The trust score calculation considers different weights: the node’s weight according to its contribution score, the node’s weight according to its cooperation times, and the weight of the distance of the research field between two nodes. Misbehaving nodes are punished based on the distance between misbehaving and requesting nodes. TrueTrust [21] calculates the service responder’s credibility as a function of all collected feedback on a service responder without using any feedback filtering mechanisms. However, the main factor in this calculation is the service requester’s feedback on a service responder, which reflects the concept of retributive justice. In [22], a system was presented that collects statistics of the participating peers’ activity in the network and defines two factors (local contribution and trustworthiness) based on the collected statistics. According to the trustworthiness factor, each peer is assigned a rank, which is then propagated to all participating peers in the network. In [23], a system using the best linear unbiased estimator was proposed that takes into account the uncertainties in the input variables to define the trust estimation method. The calculation of the trust value is based on requested and actual transfer rates and other parameters. AbsoluteTrust [24] is an algorithm that aggregates local trust without normalization. The algorithm depends on the concept of weighted averaging and scaling of local trust, which is then used to calculate the global trust. AuthenticPeer++ [25] is a hybrid technique that combines peer-based and file-based reputation system approaches. To reduce the impact of collective malicious peers, AuthenticPeer++ utilizes the global trust value of a peer to weigh its opinion. In [26], the trust calculation approach is based on direct transactions and the reputation of a set of neighbors. Each peer has a trust vector for every other peer, which stores the outcomes of the past transactions. A time-sensitive and context-dependent reputation model was developed in [27] for mobile ad hoc networks. The reputation value is built upon direct trust and recommendations from other peers according to their trust value. The cyclic ranking method was utilized in [28] for a P2P sharing system, where each peer uses its direct observation as well as recommendations collected from reputable neighbors about effective exchanges known as provision cycles. In [29], a multiagent robotic system with decentralized control was presented, where robots-agents calculate levels of trust for each other to protect the system from hidden attacks of robots-saboteurs by considering their previous interactions. The trust level of the agent is increased when the agent executes functions for the target and provides correct feedback about other robots. Trust-X [30] is a comprehensive XML-based framework for trust negotiations in a peer-to-peer network. Trust tickets are issued by involved parties after successful completion of a negotiation and used to speed up the following negotiations for the same resource.

In contrast to the large number of trust management systems that follow traditional approaches, very few systems have considered nature as a source of inspiration to target the trust problem. Here, we shed some light on them. In [31], a trust management system for P2P networks based on a genetic algorithm was presented, where the algorithm detects abnormal behavior as a function of a comparison with the peer profile. The algorithm defines the trustworthiness of peers based on anomaly detection using the behaviors of good peers as historical data, where each peer maintains a separate profile of other peers’ behaviors. The profile information is extracted from the direct interactions between peers in the network. TrustIs [32] is a trust model inspired by the human immune system (IS). It uses concepts from the body’s defense against viral attacks. This model combines peer reputation and object reputation to prevent the distribution of polluted files and the communication of malicious peers. AntRep [10,12], TACS [8], and AntPS [33] are trust and reputation trust management systems for P2P networks that rely on the ACO to find the best (most trustworthy) node. The ACO algorithm was inspired by the behavior of ant colonies [34,35,36], where a pheromone value is used to select a trustworthy provider. In AntRep [10], ants are dispatched to collect evidence about a peer’s reputation and forward it to the requester. In TACS [8], ants follow pheromone values in addition to some heuristic values to find a trustworthy provider. In AntPS [33], the selection of the trustworthy provider is based on two pheromone values: resource similarity and trust similarity. Another reputation model for P2P networks based on ACO was presented in [12], which uses pheromone values as recommendations about target peers. Table 1 shows a comparison between reviewed bioinspired trust models.

However, all of the abovementioned ant-inspired trust models were based on metaheuristic approaches and, hence, suffer from high computation overheads and long running times due to their iterative nature. In contrast, AntTrust is designed as a problem-specific heuristic to overcome these limitations. It strives to make a “good” guess for the path leading to the most trustworthy provider from the first try rather than iteratively working on optimizing a random path. Additionally, a flaw found in all ant-inspired trust models mentioned earlier is that they do not offer a way to reward good providers. Moreover, some of the models do not consider punishing misbehaving malicious nodes, except for TACS [8], which evaporates pheromones along the path to the misbehaving provider. However, this method is unfair to good nodes located along the “punished” path. AntTrust applies explicit strategies to reward and punish peers based on their trustworthiness.

## 3. Algorithm Design

### 3.1. Ant Inspiration

**Figure 1:**
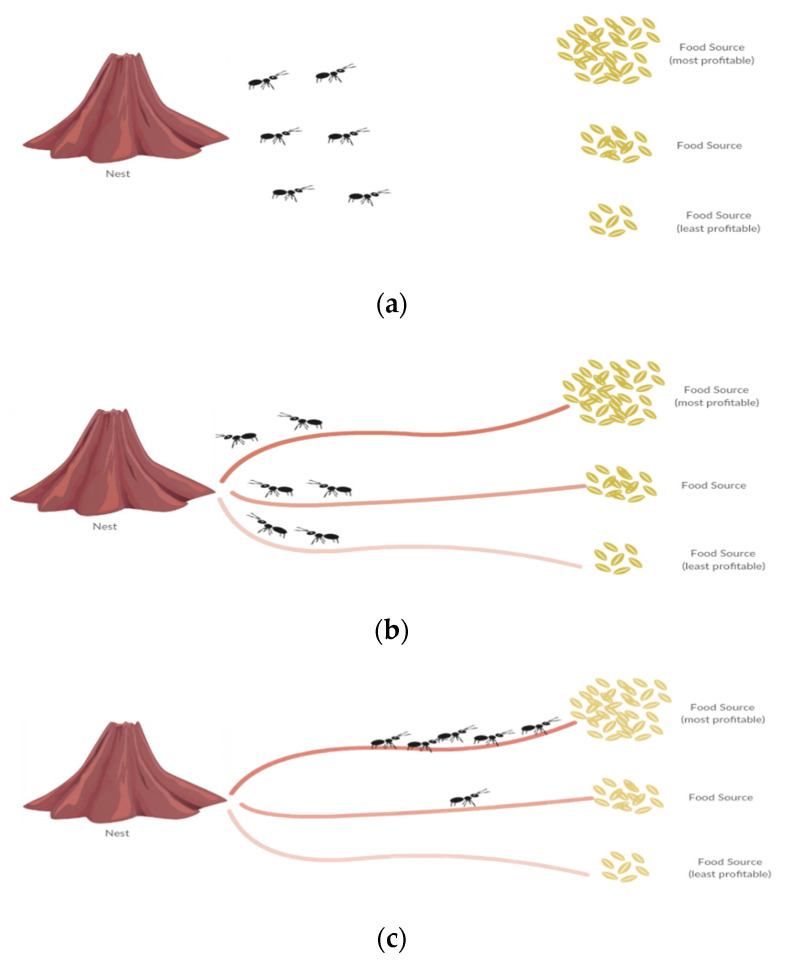
Ant foraging behavior. (**a**) With no pheromone trail in the environment, ants start exploring the environment randomly. (**b**) Ants return to the nest while depositing a pheromone trail proportional to food source profitability. (**c**) Other ants take the path that has the highest density of pheromone. The pheromone on the trails to less profitable sources evaporates gradually, while it becomes more concentrated on the path to the more profitable source.

The primary idea of AntTrust is based on ant foraging behavior. Ants start their food-foraging journey from the nest, trying to find food sources randomly around the surrounding area, as shown in Figure 1a. If an ant finds a good food source, it then assesses its profitability before going back to the nest. During its return journey, the ant releases a chemical substance (pheromone) on the ground. The density of the pheromone released by the ant is in proportion to the profitability of the food source, thereby allowing other ants to identify the path to the most profitable food sources, as shown in Figure 1b. Other ants then follow the path that has the highest density of the pheromone, as shown in Figure 1c. The pheromone evaporates over time, which leads to fewer visited paths. In sum, this behavior is a well-known example of indirect communication and is usually referred to as stigmergy [34,35,36,37,38,39].

In the AntTrust system, a file requester (which corresponds to the nest in the ant colony analogy) sends a file request (ant) to its neighboring peers (food sources). After receiving the file (reaching a food source), the requester peer gives a rating to the provider peer (pheromone deposition) based on the validity of the file received (the profitability of the food source). Furthermore, the trust value can be increased by a reward value or decreased by a punishment value depending on the validity of the received file, which mimics the idea of pheromone concentration and evaporation.

### 3.2. AntTrust System Architecture

**Figure 2:**
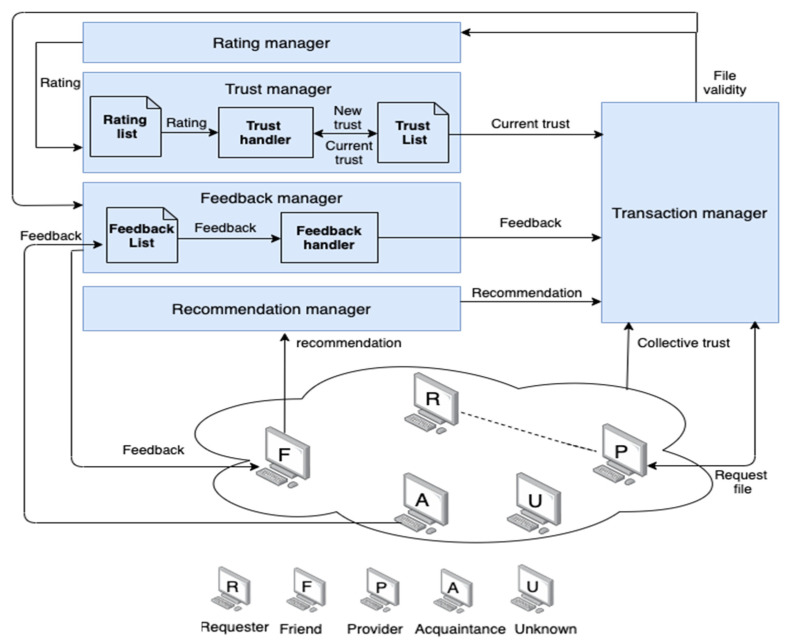
System architecture.

The underlying architecture, shown in Figure 2, is a centralized P2P network where a system registry keeps a dynamic list of all peers that joined the system at any time and a list of the files that they offer. However, the processing and calculations in the proposed trust management system are distributed where each node internally calculates the trust value based on its past communications with the other nodes. No single calculation or decision is made by a central entity. As stated earlier, the system registry serves only as a shared memory for the underlying P2P network to keep track of current available peers and resources.

Each peer maintains three local lists to support the decentralization, as described below:

- Rating list (RL): Each peer *P_i_* has an RL, which keeps records of friend IDs, the total number of positive transactions (TP), and the total number of negative transactions (TN) completed with each friend of the peer. The list is dynamically updated once the peer completes a transaction.
- Feedback list (FL): The FL is a two-dimensional matrix maintained by each peer. It keeps track of the total number of positive feedback (TP_FoA_) events and the total number of negative feedback (TN_FoA_) events according to the rating received after each transaction performed by a peer’s acquaintance with a friend of the acquaintance, for instance, if peer *P_i_* has *n* acquaintances and *m* friends of acquaintances.
- Trust list (TL): This list records the current trust (CT) values of *P_i_* for each of the peer’s friends. The trust value is calculated based on the ratings in the rating list.

The main components of the AntTrust system are as follows:

- Transaction manager: This component is responsible for selecting the most trustworthy file provider for the requester peer in each transaction by considering four trust parameters (current trust, feedback, recommendations, and collective trust). The Transaction manager receives the current trust located in the trust list (TL) and uses it to select the file provider peer. In the absence of current trust, the selection depends on the recommendation received from the Recommendation manager. In the latter case, feedback (the value received from the Feedback manager) is used to help select a provider peer. If all three of these values are unavailable, the Transaction manager uses collective trust to select a trustworthy file provider. After selecting a trustworthy file provider, the transaction takes place between the requester and the selected file provider. The requester peer then evaluates the received file and submits the file’s validity to the Rating manager and the Feedback manager.
- Rating manager: This component submits either a positive (1) or negative (0) rating for a transaction. These ratings are based on the validity of the received file from the Transaction manager.
- Trust manager: This is the main component in the system. It is responsible for calculating the new trust after each transaction as a function of assigned reward or punishment values. Our system is proactive because it calculates trust after each transaction; thus, there is no need to compute trust before the transaction, which reduces the time taken to calculate trust before any transaction. The calculation of the new trust value differs as a function of the validity of the file received. If the file is valid, the calculation depends on the total number of positive (TP) ratings and the total number of negative (TN) ratings received from the rating list (RL) as a reward to the file provider. If the file is invalid, the calculation is based on subtracting a punishment from the current trust value. The component then records the calculated new trust in the trust list (TL).
- Feedback manager: This component is responsible for exchanging ratings and feedbacks. The feedback list (FL) is used to record the received feedback from acquaintances in a list. The feedback handler is responsible for calculating the feedback using information that is received from the FL whenever necessary as a function of the total number of instances of positive feedback and negative feedback.
- Recommendation manager: This component is responsible for retrieving recommendations about a specific file provider from a friend with current trust values above the best-friend threshold (*δ_f_*), where *δ_f_* is a positive real number.

### 3.3. Definitions

The AntTrust system defines the following terms in the trust context to ease the understanding of the relationship between peers in the network:

- Requester (*P_q_*): A peer that requests a file in a transaction.
- Provider (*P_v_*): A peer that has the requested file in a transaction.
- Friend: A peer *P_j_* is a friend of peer *P_i_* if *P_i_* has previously received a file or recommendation from *P_j_*. The friendship relationship is transitive for a friend of a friend (FoF) and a friend of a friend of a friend (FoFoF). For simplicity, we assume a maximum friend chain length of three.
- Best friend: A peer *P_j_* is a best friend of peer *P_i_* if the current trust of *P_i_* by *P_j_* is above *δ_f_*.
- Acquaintance: A peer *P_j_* is an cquaintance of peer *P_i_* if *P_i_* has previously sent a file to *P_j_*.
- Unknown peer: A peer *P_j_* is unknown to peer *P_i_* if *P_i_* has not previously received a file, recommendation, or feedback from *P_j_* (it is neither a friend nor an acquaintance).
- Current trust: This is the last saved trust value in the trust list (TL). During system initialization, all current trust values are set to zero.

### 3.4. How AntTrust Works

This section explains the two algorithms used in the AntTrust system in detail: the main AntTrust algorithm and the sub-algorithm named File provider selection algorithm.

#### 3.4.1. AntTrust Algorithm

The AntTrust algorithm, as shown in Algorithm 1, has three main parts: file provider selection, file validation, and file provider recommendation, as described below.

(a) File provider selection: Initially, when all peers P in the network have no experience, *P_q_* selects *P_v_* randomly. However, after some transactions have been processed, a list of friends and their current trust values can be generated, and the provider selection process is then based on the file provider selection algorithm presented in Section 3.4.2.
(b) File validation: After *P_q_* receives a file from *P_v_*, *P_q_* rates the transaction based on the validity of the received file based on Equation (1).

(1) $$\mathrm{Rating}=\{\begin{array}{c} \;1\;\mathrm{if the received file is valid}\\ 0\;\mathrm{otherwise} \end{array}.$$

(c) File provider recommendation: Consequently, *P_q_* evaluates *P_v_* according to the validity of the received file or the received recommendations. If the file and recommendations are valid, a reward is calculated according to Equation (2) for *P_v_* and all recommender friends *F_i_*, if any, between *P_q_* and *P_v_*.

(2) $$Reward=\left(1-\left(\frac{1}{N_{t}-1}\;{\left(TP-\mu \right)}^{2}\right)\right),$$

where *N_t_* is the total number of positive and negative transactions in which *P_v_* provided a file to *P_q_*, and *N_t_* ≠ 1. TP is the total number of positive transactions in which *P_v_* provided a file to *P_q_*, and μ is the mean of all ratings of *P_v_* by *P_q_* or *F_i_*.

The proposed reward formula focuses on the normalized ratio of the positive ratings and total ratings of the provider that are given by the requester and its friends. Based on this reward value and the current trust value, a new trust value is calculated according to Equation (3):

(3) $$New\;Trust=\frac{Reward}{1+\left(Reward-CurrentTrust\right)}.$$

Afterward, CurrentTrust is updated by the *NewTrust* value, and *P_v_* is added to the *P_q_* trust list.

Conversely, if the file or recommendation is invalid, punishment and a new trust value are calculated according to Equations (4) and (5), respectively, for *P_v_* and the recommender.

(4) Punishment=PunishmentRate*CurrentTrust,

where *PunishmentRate* is an experimentally set variable. A higher value results in a more aggressive system toward invalid transactions, even by good peers.

(5) $$New\;Trust=\frac{Punishment}{1+\left(Punishment-CurrentTrust\right)}.$$

Then, CurrentTrust is updated by the *NewTrust* value.
**Algorithm 1:** AntTrust **Data:** Rating List (RL), Trust List (TL) **Result:** send a file to *P_q_*, update RL and TL **while** num of transaction <= max num of transaction do // 1. File provider selection **if** *P_q_* request a file **then** **if** *P_q_* has previous experience with any *P_i_* **then**
Select *P_v_* based on File provider selection algorithm **else** Select *P_v_* randomly // 2. File validation **if** accepted file is valid **then**
Rating= 1 // 3. File provider recommendation **if** *P_v_* recommended by *F_i_* **then** Calculate Reward and Trust value of all recommended *F_i_* between the *P_q_* and *P_v_*
Calculate Reward and Trust value of *P_v_*
**else**
Rating = 0 **if** *P_v_* recommended by *F_i_* **then** Calculate the Punishment and Trust value of *F_i_*
Calculate the Punishment and Trust value of *P_v_*
Send feedback to friends **else** Go to step 1 **end while**


#### 3.4.2. File Provider Selection Algorithm

The file provider selection algorithm, as shown in Algorithm 2, locates a trustworthy file provider *P_v_* based on four consecutive trust factors: current trust, recommendation, feedback, and collective trust. For each factor, there are three scenarios: when more than one provider satisfies the factor, when only one provider satisfies the factor, and when no provider satisfies the factor. Below, an explanation of the algorithm is presented in detail.
**Algorithm 2:** File provider selection **Data:** Trust List (TL), Feedback List (FL) **Result:** select trustworthy provider // Step 1: check Trust list **if** the number of direct friend providers > 1 **then**
**if** max Trust value > *δ_t_* **then** accept the file from *P_v_* **else** request and calculate Recommendation from friends with trust value> *δ_f_* **else if** the number of direct friend providers = 1 **then**
**if** Trust value > *δ_t_* **then** accept the file from *P_v_*
**else** deny transaction **else** request and calculate Recommendation from friends with trust value > *δ_f_* // Step 2: check Recommendation **if** the number of recommendation > 1 **then**
**if** max Recommendation > *δ_t_* **then** accept the file from *P_v_*
**else** calculate Feedback **else if** the number of recommendation = 1 **then**
**if** Recommendation> *δ_t_* **then** accept the file from *P_v_*
**else** deny transaction  ** else** calculate Feedback // Step 3: check Feedback **if** the number of Feedback >1 **then**
**if** max feedback> *δ_t_*
**then** accept the file from *P_v_*
**else** collect Trust value from all friends **else if** the number of Feedback = 1 **then**
**if** feedback > *δ_t_* **then** accept the file from *P_v_*
**else** deny transaction **else** collect Trust value from all friends // Step 4: check Collective trust **if** the number of Collective trust > 1 **then**
**if** max collective trust > *δ_t_* **then** accept the file from *P_v_* **else** select a random *P_v_* **else if** the number of Collective trust = 1 **then**
**if** Collective trust > *δ_t_* **then** accept the file from *P_v_*
**else** deny transaction **else** select a random *P_v_*

- Step 1: Based on Current Trust

When *P_q_* requests a file and more than one *P_v_* is available, *P_q_* checks its trust list first and selects a *P_v_* with a current trust value above the minimum trust threshold (*δ_t_*); otherwise, *P_q_* sends a recommendation to its friends, as described in Step 2.

When only one direct *P_v_* exists, *P_q_* should select that *P_v_* only if its current value is above *δ_t_*; otherwise, the transaction should be rejected. When no *P_v_* exist, *P_q_* sends a recommendation request to its friends.

- Step 2: Based on Recommendation

The recommendation request from *P_q_* is propagated through the friend chain until it reaches a friend *F_i_* that has direct experience with *P_v_*. However, only recommendations from best friends are considered. Recommendations are calculated according to Equation (6).

(6) $$Recommendation=CurrentTrust_{PqF1}*\ldots *CurrentTrust_{F_{N_{c}}p_{v}},$$

where *N_c_* is the number of friends in the friend chain between *P_q_* and *P_v_* with a *CurrentTrust* value greater than *δ_f_*. $CurrentTrust_{PqF1}$ is the current trust of requester peer *P_q_* for the first friend *F*_1_ in the friend chain between *P_q_* and *P_v_*, and $CurrentTrust_{F_{N_{c}}p_{v}}$ is the current trust of *P_c_*, i.e., the last friend in the friend chain between *P_q_* and *P_v_*, for the provider peer *P_v_*.

After calculating the recommendations, when *more than one recommendation* exists, *P_q_* checks the calculated recommendations and selects a *P_v_* with a recommendation above *δ_t_*; otherwise, feedback is calculated, as described in Step 3.

When only *one recommendation* exists, *P_q_* should select *P_v_* if its recommendation is above *δ_t_*; otherwise, the transaction should be rejected. When *no recommendations* exist, feedback is calculated.

- Step 3: Based on Feedback

When no recommendation is available, *P_q_* calculates feedback about *P_v_* based on the ratings received by *P_i_* from all acquaintances of *P_q_*. Feedback is calculated according to Equation (7).

(7) $$Feedback=1-\left(\frac{\frac{1}{N_{f}-1}\;{\left(FP-\mu \right)}^{2}}{1+\left(1-\left(\frac{1}{N_{f}-1}\;{\left(FP-\mu \right)}^{2}-CurrentTrust_{\;PqPv}\right)\right)}\right),$$

where *N_f_* ≠ 1, and it represents the total number of instances of positive and negative feedback provided for *P_v_* from all acquaintances of *P_q_*, *FP* is the total number of instances of positive feedback provided for *P_v_* from all acquaintances of *P_q_*, μ is the mean of all received feedback for *P_v_* from *P_i_*, and $CurrentTrust_{\;PqPv}$ is the current trust of *P_q_* for *P_v._*

After calculating the feedback, when more than one feedback is available, *P_q_* checks the calculated feedback and selects a *P_v_* with a feedback value above *δ_t_*; otherwise, a collective trust is calculated, as described in Step 4.

However, when only one feedback exists, *P_q_* should select *P_v_* only if its feedback value is above *δ_t_*; otherwise, the transaction should be rejected. If *no feedback* exists, a collective trust is calculated.

- Step 4: Based on Collective Trust

The last factor is collective trust, which is the summation of the current trust values collected from all *F_i_* in the friend chain between *P_q_* and *P_v_*, calculated according to Equation (8).

(8) $$CollectiveTrust=CurrentTrust_{F1p_{v}}+\ldots +CurrentTrust_{F_{N_{c}}p_{v}}.$$

After calculating the collective trust value, when *more than one collective trust* value exists, *P_q_* checks the calculated collective trust values and selects a *P_v_* with a collective trust value above *δ_t_*; otherwise, *P_v_* is selected randomly.

However, when *only one collective trust* value exists, *P_q_* should select *P_v_* only if its collective trust value is above *δ_t_*; otherwise, the transaction should be rejected. When *no collective trust* values exist, *P_v_* is selected randomly.

A close inspection of Algorithms 1 and 2 reveals that neither algorithm includes any nested loops or recursive calls; hence, they have a linear running time. Accordingly, the time complexity of AntTrust is O(n), which indicates high scalability and is well aligned with the experimental results presented in Section 5. On the other hand, the time complexities of EigenTrust, TACS, and TNA-SL are O(n^2^) [40], O(n^3^) [8], and O(2n) [16], respectively.

## 4. Evaluation Framework

We implemented the proposed algorithm using the simulation tool QTM using an approach similar to [17,32,41], as QTM was developed specifically for evaluating trust management systems in P2P networks. We controlled the experiments by varying the percentage of the malicious file providers, the number of transactions, and the size of the network. AntTrust considers two malicious strategies: naïve and collective. The size of the network varied in the range [132...512]. We considered two different network loads: 2500 and 5000 transactions. For each number of transactions, the percentage of malicious providers varied in the range [20%...60%]. The value of *PunishmentRate* was constant at 0.15. The considered threshold values were *δ_t_* = 0 and *δ_f_* = 0.5. Values of both thresholds were determined empirically over several runs. The benchmarks considered in the evaluation were TACS [8], EigenTrust [9], and TNA-SL [16].

All experiments were run on a SANAM cluster computer [42,43]. Each simulation scenario was executed 10 times for AntTrust and EigenTrust [9], whereas for TACS [8] and TNA-SL [16], the simulation was run once because of the overhead demanded to run such algorithms.

In a similar approach to [8], the TACS parameters used in the experiments were as follows: alpha and beta = 1, initial pheromone = 0.4928, number of iterations = 3, number of ants = 4, punishment threshold = 0.6806, path length factor = 0.5651, and transition threshold = 0.4972.

Two performance metrics were used to test the effectiveness of the developed algorithm: the success rate and execution time. The success rate is important for testing the effectiveness of the delivered algorithm in terms of providing the selection of a good provider. The equation divides the valid files received by good peers by the total number of transactions made by good peers according to Equation (9).

(9) $$\mathrm{Success}\;\mathrm{rate}=\frac{\#\;\mathrm{of}\;\mathrm{valid}\;\mathrm{files}\;\mathrm{received}\;\mathrm{by}\;\mathrm{good}\;\mathrm{peers}}{\#\;\mathrm{of}\;\mathrm{transactions}\;\mathrm{completed}\;\mathrm{by}\;\mathrm{good}\;\mathrm{peers}}.$$

The execution time (running time) represents the time taken to execute the algorithm and should be minimized. Given the importance of this measure, we aimed to show how fast AntTrust is. This measure distinguishes the AntTrust algorithm (problem-specific heuristic) from metaheuristic algorithms because it generates an efficient solution in terms of time.

## 5. Results and Discussions

As mentioned before, only a few studies have focused on reputation and trust management systems in P2P networks by employing bioinspired heuristics. To the best of our knowledge, all of the proposed bioinspired trust and management algorithms were based on metaheuristics rather than problem-specific heuristics. We compared AntTrust with EigenTrust [9], TACS [8], and TNA-SL [16]. A no-trust system (None), where the selection of a file provider was random, was used as the baseline case.

### 5.1. Success Rate

The success rate is calculated as the total number of valid files received by good peers divided by the total number of files received by good peers.

In Table 2 and Table 3, the success rate is presented against the percentage of malicious peers (naïve and collective) as the number of transactions increased from 2500 to 5000, respectively, for AntTrust and the benchmark algorithms, TACS, EigenTrust, and TNA-SL, and the baseline scenario, None.

When the number of transactions was 2500 and the number of peers was 132, and the percentage of collective malicious providers increased to 60%, the success rates of TACS, EigenTrust, TNL-SL, and None dropped significantly; TNL-SL converged with EigenTrust, attaining a success rate of around 70%, while it reached 60% in TACS. On the other hand, the success rate of AntTrust remained stable (≥91%) in both naïve and collective strategies, and regardless of the number of providers. As expected, the no-trust system produced the lowest success rate and was significantly impacted by the increasing percentage of malicious peers.

The variations in the success rate of AntTrust and the benchmark algorithms were more evident as the number of transactions was increased to 5000. AntTrust exhibited a high success rate (≥90%) in all experiments. However, the success rate of other algorithms decreased gradually to 60% in TNA-SL and approximately 70% in EigenTrust when the percentage of naive malicious peers was 60% and the number of providers was 512. For heavy scenarios of a large number of peers or transactions, we were unable to calculate the success rates of TNA-SL and TACS algorithms, as their running times exceeded 24 h.

Overall, we can observe that AntTrust outperformed the other algorithms in all scenarios. In AntTrust, with an increasing number of transactions, the requester gains more experience and more friends, which helps the algorithm in identifying the best (most trustworthy) provider. AntTrust is based on four trust components instead of selecting the provider based on aggregated trust values, as in EigenTrust, or based on a trust value (pheromone), as in TACS.

### 5.2. Execution Time

In Table 4 and Table 5, the running time is presented against the percentage of malicious peers (naïve and collective) as the number of transactions increased from 2500 to 5000, respectively, for AntTrust and the benchmark algorithms, TACS, EigenTrust, and TNA-SL, and the baseline scenario, None. The None system took the least time to execute in both strategies, which is reasonable because it randomly selects the provider. Next were AntTrust and EigenTrust, while TACS took the longest time to execute due to its nature.

As the number of transactions increased to 5000, EigenTrust exhibited slightly better performance than AntTrust when using the naïve strategy. However, this was not the case with the collective strategy, where AntTrust outperformed EigenTrust. TNA-SL with collective pure malicious peers and TACS algorithms presented a significantly high running time exceeding 24 h.

The empirical running time results align with the asymptotic complexity analyses of AntTrust and EigenTrust. However, although the asymptotic complexity analysis suggests that TNA-SL is the most complex among the studied algorithms, when implemented, TACS was the worst. This might be related to the fact that TNA-SL displays an extended running time only for lengthy FoF chains, as complex matrix chain multiplications are needed, which is not a very common scenario.

## 6. Conclusions

This paper presents a trust management system named AntTrust that is based on a problem-specific heuristic inspired by ant colonies. The goal of our system is to increase the success rate of good peers and reduce the execution running time by adopting a new algorithm that measures trust between peers in P2P networks. The main AntTrust algorithm rates the transaction on the basis of the validity of the received file and applies explicit strategies to reward and punish peers as a function of their trustworthiness. Another sub-algorithm, named the file provider selection algorithm, locates a trustworthy file provider on the basis of four consecutive trust factors (current trust, recommendation, feedback, and collective trust).

Evaluation of our system through a comprehensive framework showed that AntTrust has a strong positive effect in terms of providing a more trustful environment by increasing the success rate of good peers. Regarding the tested malicious strategies, AntTrust showed considerable ability, compared to the other algorithms, to distinguish the collective strategy from the naïve strategy due to its multiplicity of trust parameters and the punishment process for misbehaving malicious nodes. Additionally, AntTrust showed a low running time, which can be attributed to its proactivity in immediately calculating trust rather than waiting for a new transaction.

The results presented herein open a path toward research on various interesting issues. Future studies should assess whether trustworthiness on some nodes converges and stabilizes after some rounds. It is also important to consider a strategy to reward or punish an acquaintance based on the honesty of its provided feedback. In addition, the system can be tested against other malicious models, such as strategic deception. Furthermore, a special strategy can be adopted to handle the problem of multiple responses arising from the same remote friend via multiple possible chains of friends, which might occur as a result of trust propagation through a network. Lastly, future research should look at extending the application domain to other platforms, such as cloud computing, grid computing, and WSNs.

## Figures and Tables

**Table 1:** Comparison between reviewed bioinspired trust models.

System	Trust Evaluation	Bioinspiration	Malicious Models	Performance Measures	Evaluation Tool
Peer profile-based trust [31]	Detect abnormal behavior using anomaly detector based on profile	GA	Selfish peers, traitor attack, Sybil attack, collusion attack	Number of rules Number of transactions	N/A
TrustIs [32]	Detect malicious peer based on peer reputation, object reputation, and index reputation received from an immune peer	IS	Pure malicious	Normal file upload rate Polluted file filtered rate	PeerSim
AntRep [10]	ACO	AC	N/A	Hop count Success rate	FreeNet simulator
TACS [8]	ACO	AC	N/A	Percentage of selected trustworthy servers	Custom Java-based simulator
ACO-based reputation [12]	ACO and combining trust values from different paths	AC	Strategic deception, malicious collusion	Success rate Number of messages	PeerSim
AntPS [33]	ACO with a combination of trust similarity and resource similarity	AC	N/A	Request response ratio Malicious file downloads	Query Cycle Simulator

**Table 2:** The success rate for 2500 transactions.

Malicious %	AntTrust		EigenTrust		None		TNA-SL		TACS	
	Naive	Collective	Naive	Collective	Naive	Collective	Naive	Collective	Naive	Collective
**132 peers**										
20	93.57	92.13	92.32	85.83	76.95	57.09	84.00	82.98	91.29	90.08
40	91.56	94.42	72.80	90.08	40.15	77.02	74.40	75.45	81.23	80.17
60	92.78	92.57	73.68	72.85	57.17	41.39	60.53	69.82	61.68	60.53
**256 peers**										
20	94.92	94.88	90.37	81.37	76.27	58.96	83.53	84.50	*	*
40	92.69	96.25	82.62	91.42	39.29	76.23	69.18	70.28	*	*
60	95.04	94.07	70.20	69.94	58.88	40.81	60.71	59.92	*	*
**512 peers**										
20	92.71	92.98	89.90	81.74	77.59	58.59	81.65	84.32	*	*
40	94.47	95.89	82.88	88.75	40.77	77.40	73.33	71.41	*	*
60	94.16	96.13	75.98	70.08	58.21	41.06	64.68	60.24	*	*

* Running time exceeds 24 h.

**Table 3:** The success rate for 5000 transactions.

Malicious %	AntTrust		EigenTrust		None		TNA-SL		TACS	
	Naive	Collective	Naive	Collective	Naive	Collective	Naive	Collective	Naive	Collective
**132 peers**										
20	95.26	93.76	94.07	92.81	76.89	77.11	83.25	*	*	*
40	93.62	94.22	87.81	85.21	57.30	58.28	72.13	*	*	*
60	89.28	91.88	74.49	69.22	39.88	39.83	57.59	*	*	*
**256 peers**										
20	94.66	95.60	93.55	92.41	76.72	77.17	84.27	*	*	*
40	94.06	93.72	87.67	82.33	58.19	57.99	69.95	*	*	*
60	94.02	93.69	62.86	72.84	40.19	39.49	62.39	*	*	*
**512 peers**										
20	95.25	93.89	93.15	90.84	76.93	77.07	82.60	*	*	*
40	95.11	94.90	84.35	85.47	58.56	59.01	71.38	*	*	*
60	94.31	95.03	71.06	70.76	40.39	40.63	60.88	*	*	*

* Runtime exceeds 24 h.

**Table 4:** Running time for 2500 transactions.

Malicious %	AntTrust		EigenTrust		None		TNA-SL		TACS	
	Naive	Collective	Naive	Collective	Naive	Collective	Naive	Collective	Naive	Collective
**132 peers**										
20	1.49	1.48	0.92	0.90	0.52	0.52	1669.33	1228.88	59,679.53	56,978.25
40	1.48	1.43	0.90	4.38	0.51	0.59	546.72	941.26	52,061.91	52,918.49
60	1.98	2.76	0.93	32.65	0.80	1.17	626.48	20,793.53	61,600.76	53,821.82
**256 peers**										
20	4.15	4.19	2.29	2.53	1.06	1.05	9594.23	9119.80	*	*
40	4.17	4.39	2.22	33.19	1.03	1.87	3254.11	15,212.95	*	*
60	7.26	12.62	2.35	276.89	4.46	9.39	7501.21	16,774.92	*	*
**512 peers**										
20	18.19	19.05	10.66	10.35	5.02	4.92	20,816.17	58,293.70	*	*
40	19.15	21.17	9.99	254.18	5.04	9.13	28,986.47	73,275.82	*	*
60	30.41	49.67	10.47	2293.28	21.83	41.85	25,337.93	78,108.66	*	*

* Running time exceeds 24 h.

**Table 5:** Running time for 5000 transactions.

Malicious %	AntTrust		EigenTrust		None		TNA-SL		TACS	
	Naive	Collective	Naive	Collective	Naive	Collective	Naive	Collective	Naive	Collective
**132 peers**										
20	1.94	2.27	1.28	8.45	0.74	0.91	9041.24	*	*	*
40	2.22	3.25	1.27	31.71	0.73	1.48	21,101.89	*	*	*
60	2.29	4.92	1.32	69.02	0.69	2.24	33,176.29	*	*	*
**256 peers**										
20	7.26	8.09	4.66	62.00	1.61	3.96	24,619.67	*	*	*
40	7.42	13.71	4.59	248.70	1.56	9.98	28,800.96	*	*	*
60	7.91	23.83	4.29	574.24	1.59	21.73	36,182.73	*	*	*
**512 peers**										
20	31.87	36.51	19.43	522.13	8.74	15.10	70,962.73	*	*	*
40	34.03	60.32	19.37	2113.92	7.30	39.16	66,033.12	*	*	*
60	36.58	97.35	19.15	4750.10	7.33	80.78	72,107.74	*	*	*

* Running time exceeds 24 h.
